# Laterally Driven Resonant Pressure Sensor with Etched Silicon Dual Diaphragms and Combined Beams

**DOI:** 10.3390/s16020158

**Published:** 2016-01-26

**Authors:** Xiaohui Du, Yifang Liu, Anlin Li, Zhou Zhou, Daoheng Sun, Lingyun Wang

**Affiliations:** Department of Mechanical and Electrical Engineering, Xiamen University, Xiamen 361005, China; duxiaohui@stu.xmu.edu.cn (X.D.); yfliu@xmu.edu.cn (Y.L.); lianlin@stu.xmu.edu.cn (A.L.); zhouzhou@stu.xmu.edu.cn (Z.Z.)

**Keywords:** resonant pressure sensor, dual diaphragms, mechanical mechanism, combined beams, high precision, microelectromechanical systems (MEMS)

## Abstract

A novel structure of the resonant pressure sensor is presented in this paper, which tactfully employs intercoupling between dual pressure-sensing diaphragms and a laterally driven resonant strain gauge. After the resonant pressure sensor principle is introduced, the coupling mechanism of the diaphragms and resonator is analyzed and the frequency equation of the resonator based on the triangle geometry theory is developed for this new coupling structure. The finite element (FE) simulation results match the theoretical analysis over the full scale of the device. This pressure sensor was first fabricated by dry/wet etching and thermal silicon bonding, followed by vacuum-packaging using anodic bonding technology. The test maximum error of the fabricated sensor is 0.0310%F.S. (full scale) in the range of 30 to 190 kPa, its pressure sensitivity is negative and exceeding 8 Hz/kPa, and its Q-factor reaches 20,000 after wafer vacuum-packaging. A novel resonant pressure sensor with high accuracy is presented in this paper.

## 1. Introduction

The resonant pressure sensor, which is one of the typical microelectromechanical systems (MEMS) devices, has been successfully applied in aerospace, industry control, and instrument calibration due to its highly accurate frequency output. In the past 30 years, several different structures of resonant pressure sensors with different excitation and detection types have been developed [1,2,3,4,5,6]. Depending on the coupling mechanisms between pressure and resonator, these pressure sensors can be classified into vibrating diaphragm structure and diaphragm/beam coupling structure. The coupling structure has a simpler structure function partition, which can easily be divided into two connected parts: the pressure-sensing element and the pressure transition element. As is well known, a diaphragm, which has been widely used in capacitive and piezoresistive pressure sensors, is the most common sensing element. A resonator with a high quality factor (Q-factor) [7], whose resonant frequency is changed with the beam internal stress, is used as a pressure transition element.

A critical design element for a MEMS pressure sensor is to maximize the conversion from the pressure-induced diaphragm strain to the resonator strain while maintaining minimal un-wanted mechanical coupling between the diaphragm and the resonator with a limited design size, which define the sensor size, Q-factor, pressure full-scale range, linearity, and frequency stability. Several structures have been tried to minimize the mechanical coupling between the diaphragm and the resonator. One of the most effective structures is that the direction of the fundamental resonant modes of the diagram and the resonator are perpendicular [8,9,10,11]. Generally, the deflection direction of the diaphragm is in the out-of-plane and the vibration direction of the resonator is in the in-plane. However, the deflection of the diaphragm can make the resonant beam shift and bend in the out-of-plane direction if the resonator is placed on the same diaphragm as the pressured-induced diaphragm [1,8,12]. In this way, the converted strain from the diaphragm to the resonator is not pure axial beam stress. For the capacitive sensing resonator, this phenomenon would cause the capacitance change not only in the in-plane direction but also in the out-of-plane direction. The in-plane capacitance change is employed for the resonant mode, while the pressured-induced out-of-plane capacitance change will lead to the phase shift in the self-exciting oscillation circuit. As a consequence, the out-of-plane capacitance change will introduce phase noise in the circuit and reduce the measuring range of the sensor. When the phase shift exceeds the self-oscillation phase range, the circuit will freeze the oscillator.

In this paper, a pressure sensor with dual diaphragms and combined beams to achieve a purer structure function partition and reduce the unnecessary coupling between the diaphragms and the resonator is presented. Structure design, finite element simulation, fabrication, and test results are included in this paper.

## 2. Sensor Design

**Figure 1:**
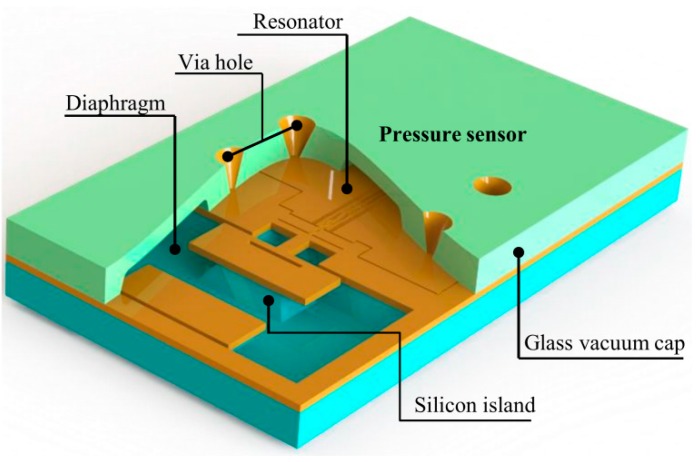
Schematic of the proposed laterally driven resonant pressure sensor without electrodes. The blue body represents pressure-sensing layer with dual symmetrical diaphragms, the yellow body represents resonator layer, and the green body represents vacuum cap layer.

The sensor chip is designed with three layers and they are the pressure-sensing layer with dual symmetrical diaphragms, the resonator layer, and the vacuum cap layer with a via hole for the electric connection. A schematic of the sensor is shown in Figure 1. Each diaphragm is attached to one end of the resonator by a silicon island. Silicon fusion bonding, anodic bonding, silicon anisotropic wet etching, lapping, chemico-mechanical polishing (CMP), and deep reactive ion etching (DRIE) technologies are applied to fabricate this sensor. All the fabrication processes are expected to be achieved on a four-inch wafer level, and the detailed processes are described in the next section. The designed resonant pressure sensor, based on electrostatic-driven [13] and capacitive detection [14], has two main functionality parts: (1) the diaphragms and silicon island for force transmission; (2) the stable resonator for frequency output. The silicon island can increase the sensing sensitivity without large the out-of-plane displacement of the vibrating beams compared to the sensor fabricated with silicon on insulator (SOI) [15,16].

### 2.1. Coupling Mechanical Mechanism between Diaphragms and Resonator

**Figure 2:**
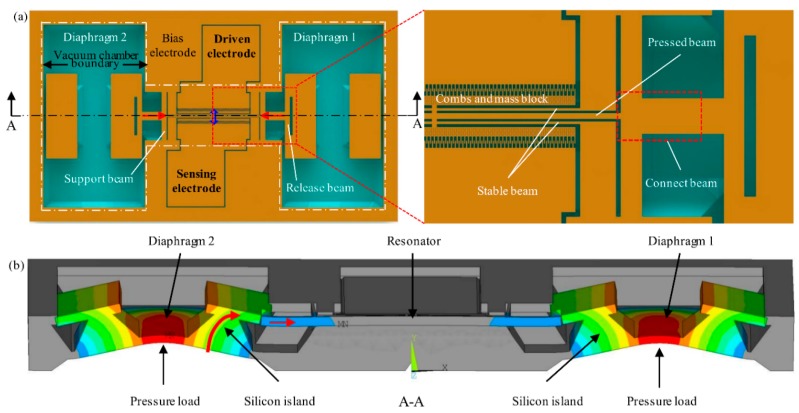
(**a**) Schematic of the sensor without glass vacuum cap for top view of the silicon resonator and force transmission from the dual diaphragms to resonator; (**b**) Section view of the structure in (**a**) for explaining the stress from the diaphragms transmitted through the closed pressure wall to the resonator.

The structure definition must be clear before theory calculation. Figure 2 shows a schematic of the silicon resonator and force transmission from the symmetrical dual diaphragms to the resonator, and the dual diaphragms are marked for clear view. As seen in Figure 2a, the resonator, which is suspended above the substrate by support beams, consists of combs and the resonant mass, pressed beam, and stable beams. The pressed beam and stable beams are the main resonant structures, and the stable beams increase the torsional stiffness and stability of the resonant beams. On one side of the fixed combs is the driven electrode and on the other side is the sensing electrode for the frequency readout, the vibration direction is seen along the blue arrow hollow, as shown in Figure 2a. Two ends of the pressed beam are connected to the silicon islands attached to the diaphragms by connect beams and release beams, and all of the beams are suspended, as shown in Figure 2b. The structure in the internal area of the dash-dot line (Figure 2a) is vacuum-packaged with a glass cap as seen in Figure 1, so the resonator vibrates with low air damping for a high Q-factor. External pressure leads to diaphragm deflection, and press stress, induced by diaphragm deflection, is applied on the ends of the pressed beam via the connect beams and release beams. The red arrow in Figure 2a represents the stress direction. Therefore, the resonant frequency of the resonator could be changed by the external pressure.

**Figure 3:**
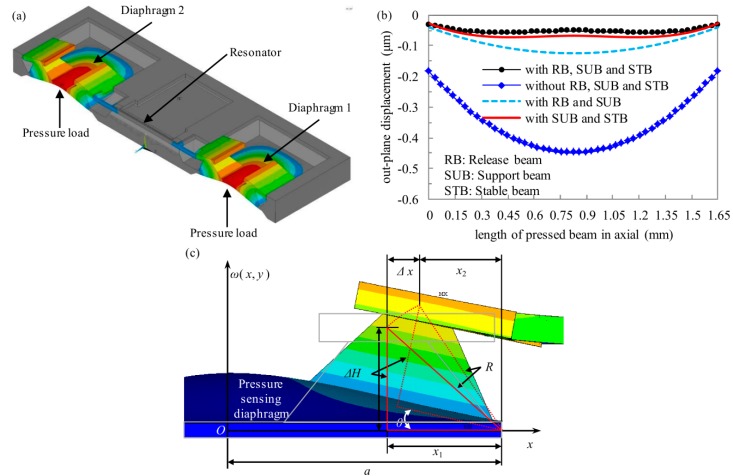
Mechanical model of the force transmission structure. (**a**) The FE displacement result when the pressure leads to deflection of the diaphragms and silicon island; (**b**) Roles of release beams, support beams, and stable beams on out-of-plane displacement of the pressed beam; (**c**) Stress transmission structure.

Figure 3a is the displacement finite element (FE) which results when pressure loads on the diaphragms, and the results indicate that there is almost no out-of-plane displacement in the resonator when pressure creates deflection of the diaphragms and silicon islands. FE simulation results about the roles of the release beams, support beams, and stable beams are shown in Figure 3b, displacement data on the bottom face of the pressed beam is obtained, and the results indicate that the support beams play the most important role in preventing the resonator from being deflected out-of-plane by the applied pressure even with the two diaphragms. The malposition between the movable combs and the fixed combs is decreased about an order of magnitude in an *ANSYS* software simulation when the release beams, support beams, and stable beams are added.

The sensor is calculated in two parts, as described at the end of the first paragraph in Section 2. In the operation of the sensor, axial displacement Δ*x* of the connect beams is the link of the two parts, and Δ*x* is also the horizontal displacement of the force transmission of the silicon island. To quantifiably analyze the effect of the diaphragms and silicon islands on the range and sensitivity of the sensor, the mechanical model is set in Figure 3c and theoretical derivation follows. Compared with the thinner pressure-sensing diaphragms, the strong silicon island can be regarded as a rigid body. The size of each diaphragm is 1500 μm × 3000 μm and silicon islands are located along the long side of the diaphragms, as seen in Figure 2. The island is deemed to rotate about the long side line when pressure is loaded, as seen in Figure 3c, since the big diaphragm has a tiny deformation [17].

According to Figure 3c and triangle geometry theory, horizontal displacement of the force transmission silicon island can be obtained from

(1) $$\Delta x=x_{1}-\sqrt{x_{1}^{2}+\Delta H^{2}}\cos\left(\theta +\arctan\left(\Delta H/x_{1}\right)\right)$$

where *x*_1_ is distance between the silicon island and the long side of each diaphragm, Δ*H* is the height of the island, *θ* is the rotation angle of the island when pressure is loaded.

The value of *θ* is small since the deformation of the big diaphragm is small, and so *θ* is approximately

(2) $$\theta \approx \arctan\left(w_{p}\left(a-x_{1},0\right)/x_{1}\right)$$

where *a* is half of the width of each diaphragm, and *w_p_*(a − *x*_1_, 0) is the deflection of the silicon island.

Thin diaphragm deformation theory is applied to calculate *w_p_*(a − *x*_1_, 0), and uniform distribution pressure is loaded on the diaphragm. The approximate solution of the trigonometric series of the small deflection fundamental equation is shown as

(3) $$w_{p}\left(x,y\right)\approx \frac{4pa^{4}}{\pi^{4}\frac{Et^{3}}{12\left(1-\mu^{4}\right)}\left(3+2\frac{a^{2}}{b^{2}}+3\frac{a^{4}}{b^{4}}\right)}\left(1+\cos\frac{\pi x}{a}\right)\left(1+\cos\frac{\pi y}{b}\right){|}_{x=a-x_{1},y=0}$$

where *p* is the pressure load, *a* and *b* are the width and length of the diaphragm, *t* is the thickness of the diaphragm, *μ* is the poisson ratio of silicon, *E* is Young’s modulus of <100> silicon.

For the very high packaging vacuum, which means low damping, the natural frequency *f* of the first mode shape can be calculated from

(4) $$\Delta x=[\left(k_{sup}l_{main}/E+A_{main}\right)/k_{tors}+l_{main}/E+\left(k_{sup}l_{main}/E+A_{main}\right)l_{con}/\left(EA_{con}\right)]\left[1-{\left(f/f_{0}\right)}^{2}\right]\sigma_{crit}$$

where *k_sup_* is the support beam stiffness in the axial direction of the pressed beam, *l_press_* and *A_press_* are the pressed beam length and cross area, *k_relea_* is the release beam stiffness in the axial direction of the pressed beam, *l_con_* and *A_con_* are the connect beam length and cross area, *f*_0_ is the resonator natural frequency when there is no pressure load, *σ_crit_* is the critical press stress.

Note that *f*_0_ is also the fundamental frequency of the pressure sensor. The vibration beam mass cannot be ignored since the mass block is not big enough, and the heavy beam model [18] is applied with concentrated mass to calculate the natural frequency; *f*_0_ can be written as

(5) $$f_{0}=\frac{1}{2\pi }\sqrt{k_{eff}/m_{eff}}=\frac{1}{2\pi }\sqrt{196EI_{main}/l_{main}^{3}(m_{mass}+0.375m_{main})+2\cdot 196EI_{limit}/l_{limit}^{3}(m_{mass}+0.375m_{limit})}$$

where *k_eff_* is the equivalent stiffness at zero axial load, *m_eff_* is the equivalent mass, *EI_press_* is the flexural stiffness of the pressed beam, *EI_stable_* is the flexural stiffness of stable beam, *l_stable_* is the length of the stable beam, *m_mass_* is the mass of the comb and mass block, *m_press_* and *m_stable_* are the mass of the pressed beam and stable beam.

Critical press stress *σ_crit_* can be inferred from the traditional bar stability theory, and the stiffness of the stable beam must be added in the deflection differential equation of the parallel combination beam [19]; hence, *σ_crit_* can be written as

(6) $$\sigma_{crit}=\pi^{2}EI_{main}/\left[{\left(\varepsilon l_{main}\right)}^{2}A_{main}\right]\cdot \left(k_{main}+2k_{limit}\right)/k_{main}$$

where *ε* is the length coefficient of the pressed beam when the beam is pressed, *k_press_* and *k_stable_* are the effective stiffness of the pressed beam and stable beam. *K_press_* and *k_stable_* can be obtained by

(7) $$k_{main}=196EI_{main}/l_{main}^{3},k_{limit}=196EI_{limit}/l_{limit}^{3}$$

Substituting Equations (2) and (3) into Equation (1), the relation of *f* and Δ*x* is built. Substituting Equations (5–7) into Equation (4), the pressure load *p* is related to Δ*x*. Thus, the pressure-frequency relation is obtained from Equations (1) and (4). Obviously, the scale factor (rate of change of the frequency of the working mode with respect to the pressure load) is not easily calculated, so the mathematic software *mathematica 4.0* is used to solve the frequency function of the pressure with different structure parameters.

### 2.2. Finite Element Simulation

**Figure 4:**
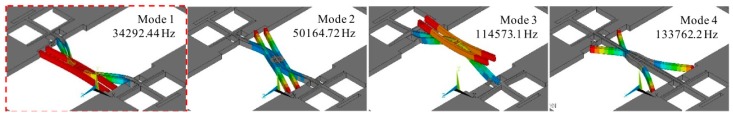
The FE simulation results of first four resonant mode shapes with no load, and the applied mode for the sensor is Mode 1.

To form a direct view of the low order mode shapes and verify the resonant theory, finite element simulation of the resonator is performed with educational ANSYS. Quadratic elements were used for increasing the calculation accuracy and the first four resonant modes are obtained in Figure 4. The working mode for the pressure sensor is the first mode (Mode 1 in Figure 4). Simulation results show that the lowest mode frequency exceeds 20 kHz which meets most aerospace applications. Frequencies of Modes 1, 3 and 4 are almost uncorrelated with the main vibration shape (Mode 1). There is no torsion mode of the pressed beam in low modes which is benefits from added stable beams.

**Figure 5:**
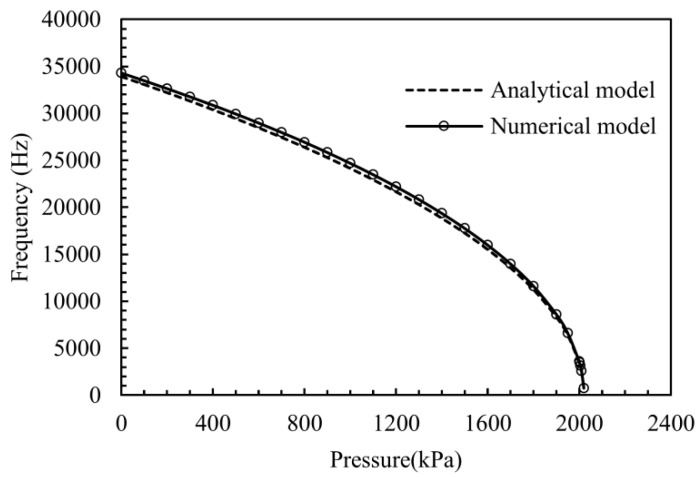
Frequency-applied pressure plot for pressure load test by calculation and FE simulation.

Natural frequency *versus* pressure loading for the first resonant mode is simulated by ANSYS, and the structure parameters are shown in Table 1. Simulation results are shown in Figure 5, where the resonant frequency of Mode 1 drops against the increased pressure, and the relationship between Mode 1 and the pressure can be well fitted by the quadratic polynomial. The range limit of the working mode is about 2000 kPa, and the sensitivity exceeds 8 Hz/kPa when the range is 0–400 kPa. The calculation result from Equations (1)–(7) is also presented in the same picture to form test feasibility of our design structure. The finite element (FE) analytical result and the numerical result are consistent with each other over the entire pressure range of the device. The compared results also indicate that immense linear relationship appears in the range of 20% limit pressure.

**Table 1:** Summary of geometric dimensions and material parameters for the sensor presented.

Quantity	Symbol	Value
Pressed beam length	*L*_press_	1650 μm
Pressed beam cross area	*A*_press_	2000 μm^2^
second moment of area of pressed beam	*I*_press_	1.0417 × 10^−19^ m^4^
Pressed beam mass	*M*_press_	7.5900 × 10^−9^ kg
Support beam stiffness	*k*_sup_	343.8574 N/m
Release beam stiffness	*K*_relea_	7141.4063 N/m
Connect beam length	*l*_con_	442.5000 μm
Connect beam cross area	*A*_con_	4000 μm^2^
Stable beam length	*L*_stable_	1308 μm
second moment of area of stable beam	*I*_stable_	8.8733 × 10^−21^ m^4^
Stable beam mass	*M*_stable_	2.6474 × 10^−9^ kg
Comb and mass block mass	*m*_mass_	1.9847 × 10^−8^ kg
Distance between island and the long side of the diaphragm	*x*_1_	305 μm
Silicon island height	Δ*H*	300 μm
Pressure sensing diaphragm thickness	*t*	40 μm
Young’s modulus of <100> silicon	*E*	165 GPa
Poisson ratio of silicon	*μ*	0.22
Length coefficient of compressive bar	*ε*	0.5

## 3. Fabrication of the Sensor and Test Scheme

### 3.1. Fabrication Process

**Figure 6:**
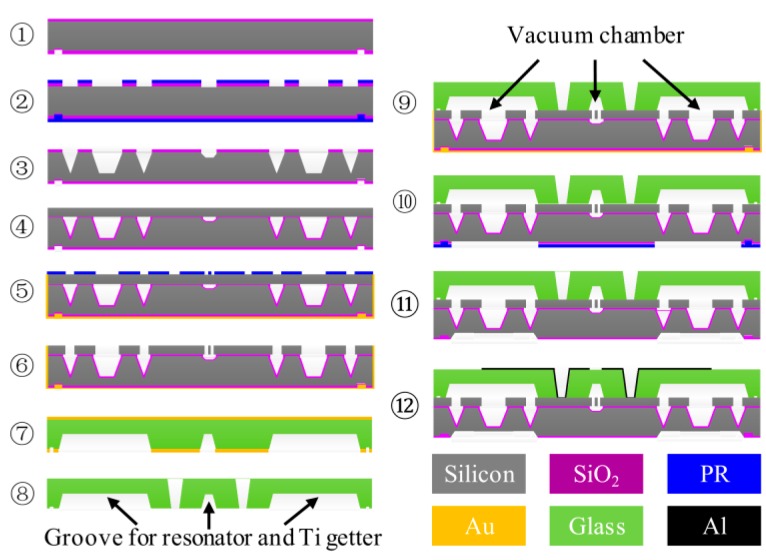
Schematic of the multilayer microfabrication approaches. Silicon fusion bonding plays a most important part in force transmission, and anodic bonding is the key step for vacuum-packaging.

**Figure 7:**
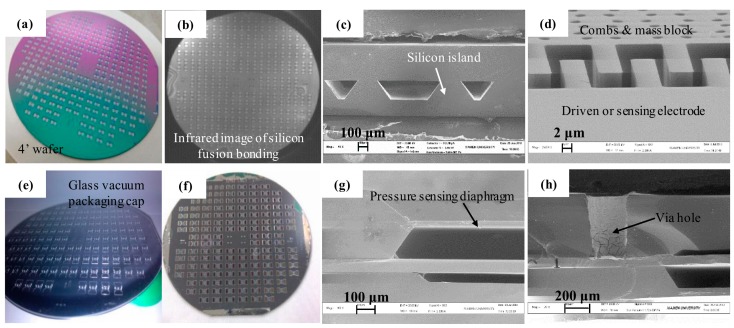
Photographs and SEM images of some key steps in the process in Figure 6. Photographs are results of: (**a**) silicon island wet etching for ③, (**b**) silicon fusion boding for ④, (**e**) via hole for ⑧, and (**f**) anodic bonding for ⑨. SEM images are results of: (**c**) silicon fusion bonding for ④, (**d**) DRIE for ⑥, (**g**) pressure-sensing diaphragms’ wet etching for ⑪, and (**h**) metal electrode in via hole for ⑫.

To verify the design theory, the resonant sensor was microfabricated with MEMS technology (Figure 6). Silicon anisotropic wet etching with tetra methyl ammonium hydroxide (TMAH), and the SiO_2_ mask were adopted to handle the pressure-sensing diaphragm and the form force transmission silicon island. Silicon fusion bonding technology was applied to attach the pressure-sensing layer and resonant layer, and the insulative layer between the two layers is 0.3 μm SiO_2_ by thermal oxidation. The 80-μm-thickness resonant layer was processed by photoresist mask and deep reactive ion etching (DRIE) after lapping and chemical mechanical polishing (CMP) to fabricate the resonator and the different beams. Pyrex 7740 glass wafer was chosen to bond the resonant layer and the glass cap for vacuum-packaging after the laser made the via hole. The diaphragms were completed by anisotropic wet etching with a back protection fixture. The driven and sensing electrodes were attached on the top surface of the glass cap by Al sputtering. The main results of the process are shown in Figure 7, and these perfect results are the guarantee of the sensor working. The silicon fusion bonding area can reach over 90%, the fabrication error of the resonant structure and diaphragm is controlled within ±1 μm and ±2 μm, respectively, and the percent of the pass of Al electrodes is approaching 100%.

### 3.2. Sensor Static Test Scheme

**Figure 8:**
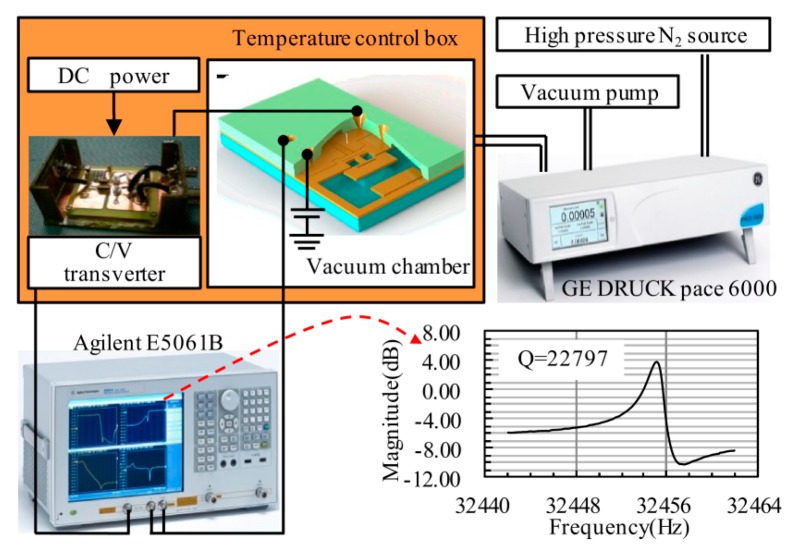
Open-loop experimental set-up for the characterization of the resonator and obtaining resonant frequency under different pressure loads.

The open-loop test frame is set in Figure 8. GE Druck PACE 6000 (0–200 kPa and 0–1 MPa, accuracy of calibration range is 0.0125%) was used for accurate pressure control, vacuum chamber and temperature controller were used to stabilize the operation environment. The driven electrode was connected to the network analyzer to drive the resonator, and the sensing electrode was connected to the network analyzer by a C/V transverter. A C/V transformation circuit was applied to transform the capacitive signal to the voltage signal, and an Agilent E5061B network analyzer was used to sweep the frequency driving and calculate the bode diagram. Gain results and Q-factor can be also calculated using software in the network analyzer. The Q-factor was obtained to evaluate the packaging vacuum and provide a basis for the circuit design. From our test results, the Q-factor of the sensor could reach 20,000, and it is enough for a closed-loop application with a shorter oscillation starting time. The high Q-factor benefited from the Ti getter and the high temperature out-gassing, and 500 nm sputtering Ti was placed on the bottom of the glass groove. Out-gassing under 400 °C for 4 h was performed before anodic bonding, and the Ti getter activating under 480 °C for 10 h was performed after anodic bonding.

## 4. Characterization Results and Discussions

**Figure 9:**
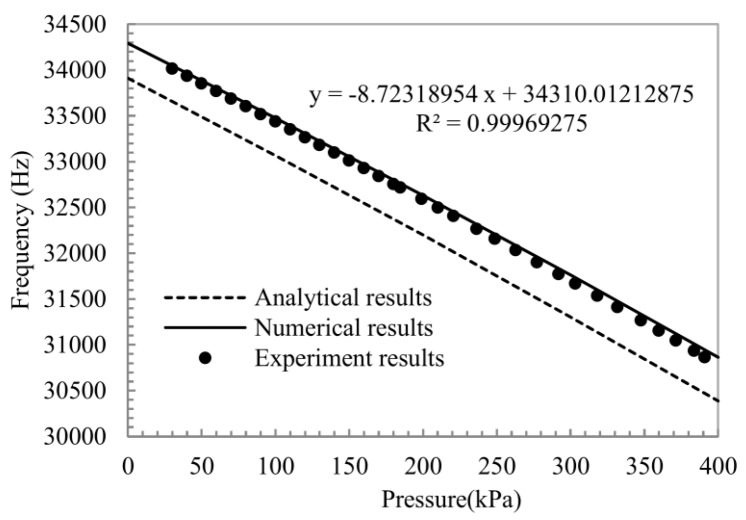
Frequency-applied pressure plot for wide pressure range test at room temperature. Each solid black dot represents one average measurement value of positive and negative strokes.

The relation of the frequency response and applied pressure (30–400 kPa) is measured at room temperature to verify the functionality of the designed sensor. Results from the numerical calculation, FE simulation and experiment are shown together in Figure 9, where the black dots represent experimental results. With the pressure controlled by an external instrument, the frequency shift is collected by the network analyzer. The analytical result is calculated by Equations (1)–(7), and the coefficient of pressure sensitivity is about −8.7 Hz/kPa over the pressure range. The measurement result of the sensor is in excellent agreement with the analytical and numerical results over the exceeding pressure range of the device. The experimental results have immense linear relationship as the analysis mentioned before.

**Figure 10:**
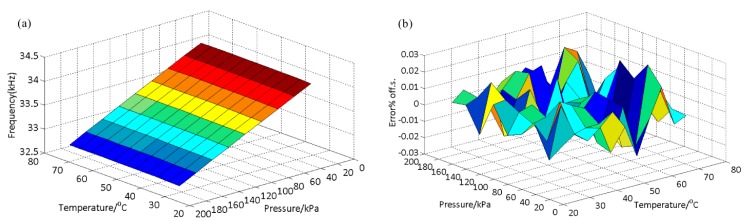
Single positive and negative strokes cycle pressure and temperature load results (**a**) and errors (**b**) (30–190 kPa and 30 °C to 75 °C). Polynomial algorithm is used for surface fitting and error calculation, the full range error is about 0.0310%F.S.

The effect of temperature on the resonant frequency must be considered since the micro-beams, micro-diaphragm, and micro-cantilevers are sensitive to temperature [20]. Thus, temperature compensation is crucial for high-precision application [21,22]. Output frequencies of the sensor with the applied pressure range (30–190 kPa) and temperature (30 °C to 75 °C) range were obtained using an open-loop test system (as seen in Figure 8). Output frequencies *versus* pressure and temperature are drawn in Figure 10a. Considering the linear coefficient of the temperature-sensing (as seen in Figure 10a), the polynomial fitting method is adopted to calibrate the pressure sensor. The calibration formula can be written as

(8) $$p_{c}=\sum_{i}^{n}\sum_{j}^{m}C_{ij}f^{i}T^{j}(n\geq 3,m\geq 2,i=0\;to\;n,j=0\;to\;m)$$

where *p_c_* is the calculated pressure, *C_ij_* is the fitting coefficient, and *T* is the calibration temperature.

In fitting, *n* = 5 and *m* = 3; *i* = 0, 1, 2, 3, 4, 5; *j* = 0, 1, 2, 3. *C_ij_* is calculated by measured frequencies, applied pressure and temperature points according to the minimum sum-of-squares of the difference values (D-values) between the calculated pressure and applied pressure. The calculation method is referred to as the least square method in Matlab.

Surface fitting based on Equation (8) with a minimum sum of the squared residual limit is performed in Matlab 2010a, and the calculation error (ratio between the D-values and the full pressure scale) is presented in Figure 10b. The maximum calculation error with the full scale (F.S.) of pressure and temperature is 0.0310%F.S., and the low error level means high measurement accuracy. The error result indicates that the proposed resonator coupled with dual diaphragms with a pure structure function partition can be used for precision pressure measurement.

**Figure 11:**
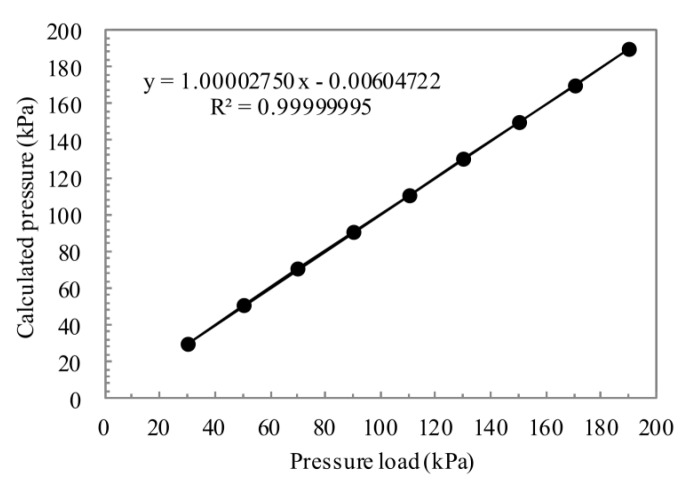
Calculated pressure-applied pressure plot for polynomial algorithm test at 30 °C.

The relation of the calculated pressure according to Equation (8) and the standard pressure is shown in Figure 11. The calibration pressure has a good linear relation with the applied pressure load over the entire pressure range (30–190 kPa) of the device. This means the pure structure function partition is available and our pressure sensor can be applied in high-accuracy measurements with the calculated coefficient *C_ij_* matrix.

## 5. Conclusions

This article demonstrates the operation of a novel structure of a resonant pressure sensor with intercoupling between the dual pressure-sensing diaphragms and a laterally driven resonator with combined beams. The coupling mechanism around the diaphragms and the combined beams has been analyzed and the frequency equation of the resonator based on the triangle geometry theory was developed for this coupling structure. FE simulation results match the theoretical analysis over the full scale of the device. Dry/wet etching, silicon fusion bonding technology, and some other MEMS technologies were used to fabricate this device, followed by vacuum-packaging using anodic bonding technology. The sensor has tested with a maximum error of 0.0310%F.S. in the range of 30–190 kPa, its pressure sensitivity is negative and exceeds 8 Hz/kPa, and its Q-factor reaches 20,000 with wafer vacuum-packaging.
